# Habitat Suitability Analysis for *Luehdorfia chinensis* Leech, 1893 (Lepidoptera: Papilionidae) in the Middle and Lower Yangtze River: A Study Based on the MaxEnt Model

**DOI:** 10.3390/insects16040396

**Published:** 2025-04-09

**Authors:** Anqi Chen, Biyu Liu, Rui Zhou, Hui Zhang, Lan Zhou, Xizhu Xie, Zhihang Zhuo, Danping Xu

**Affiliations:** College of Life Science, China West Normal University, Nanchong 637002, China; anqi2990144173@foxmail.com (A.C.); biyuliuql@foxmail.com (B.L.); zhou572922868@foxmail.com (R.Z.); zhang1612702377@foxmail.com (H.Z.); zhouzhou1731146795@foxmail.com (L.Z.); xizhu_555@foxmail.com (X.X.)

**Keywords:** *Luehdorfia chinensis*, maximum entropy model (MaxEnt), distribution, environmental variables, potential distribution centroid

## Abstract

*L. chinensis* is a National Grade II Protected Animal in China, and predicting its suitable habitats in the middle and lower reaches of the Yangtze River is crucial for developing effective conservation strategies. This study uses methods such as the MaxEnt model to predict suitable habitats for *L. chinensis* in the region, providing a scientific basis for species conservation. The model results show that the AUC value of MaxEnt is 0.989, indicating excellent predictive performance. Under current climatic conditions, the area of highly suitable habitats for *L. chinensis* in the middle and lower reaches of the Yangtze River is 75.1 × 10^3^ km^2^, accounting for 8.8% of the total area of the region. However, under the SSP2-4.5 climate scenario, the predicted results indicate a general decline in suitable habitats for the species. This change signals that the survival of *L. chinensis* will face significant risks in the future, highlighting the urgent need to develop and implement scientifically effective conservation measures.

## Simple Summary

This study aims to use the MaxEnt model to predict the distribution and changes in *Luehdorfia chinensis* (Papilionidae, Lepidoptera) under the SSP2-4.5 climate scenario. Temperature and precipitation are the main factors influencing suitable habitats for *L. chinensis*. By the 2090s, the area of suitable habitats may decrease, while the area of unsuitable habitats may increase significantly. These changes will pose significant risks to the survival of *L. chinensis*, potentially leading to increased interspecies competition, habitat loss, and a decline in population. Therefore, protecting suitable habitats for *L. chinensis* and implementing effective management in relevant areas are key measures for the conservation of this species.

## 1. Introduction

*Luehdorfia chinensis* (Leech, 1893) is an insect in the family Papilionidae and the genus *Luehdorfia* of the Order Lepidoptera. It is a rare and endemic species in China [1]. According to the IUCN Red List of Threatened Species published in 2012, *L. chinensis* is classified as a second-class national protected species in China [2]. The primary host plants of this species belong to the genus *Asarum* L., with the larvae feeding on the leaves, while the adults feed on nectar, particularly preferring the nectar of plants such as *Viola philippica* Cav. and *Taraxacum mongolicum* Hand.-Mazz [3]. It has only one generation per year. The adults emerge from their pupae in March and April, with a lifespan of about 10–15 days. They have weak flying ability and a limited dispersal range [4]. They thrive in shaded, moist environments, such as deciduous forests, where high humidity and moderate temperatures support their survival [5]. The reproductive strategy of *L. chinensis* is also noteworthy. Following emergence, *L. chinensis* undergoes a short mating phase, during which males patrol territories to locate females. Females, on the other hand, are highly selective in choosing oviposition sites, often favoring *Asarum* plants that are in optimal condition to ensure the survival of their offspring. This reproductive behavior, combined with their univoltine life cycle, makes *L. chinensis* particularly vulnerable to environmental changes and habitat disturbances [4,5]. However, habitat destruction, over-harvesting of host plants, and climate change pose severe threats to their survival. Currently, research on the distribution of *L. chinensis* in China is insufficient. Therefore, using the “appropriate tools” to predict its habitat has become an urgent and important issue that needs to be addressed [6].

Butterflies are an important indicator species for habitat quality and environmental health, being highly sensitive to habitat disturbances and environmental changes, and are significantly affected by such alterations. Factors such as climate change and human interference can profoundly impact biological systems, leading to shifts in the timing of key life history events, changes in geographic distribution and phenotypic traits, altered migration patterns, and even population extinction. Therefore, predicting changes in suitable habitats for protected species under climate change is crucial for understanding species adaptation patterns and establishing effective conservation systems for endangered species. In recent decades, species distribution models (SDMs) have been widely used to assess the impact of climate change on species distribution and predict distribution changes under different climate scenarios [7]. Commonly used SDMs include the Maximum Entropy Model (MaxEnt), Genetic Algorithm for Rule-set Production (GARP), Generalized Linear Models (GLMs), and Ecological Niche Factor Analysis (ENFA) [8]. Among these, the MaxEnt model, proposed by Steven J. Phillips in 2004, is a species distribution model based on the principle of maximum entropy [9]. It integrates known species distribution data with environmental variables to infer the optimal distribution state under specific niche constraints [10]. The core principle of MaxEnt is to predict the potential distribution range of species under maximum entropy conditions [11]. The model is particularly effective in handling small sample sizes and testing multiple variables [12], making it highly suitable for predicting suitable habitats for *L. chinensis* in this study.

This study aims to predict the suitable habitat distribution of *L. chinensis* in the middle and lower reaches of the Yangtze River using the MaxEnt model. By integrating species occurrence records and environmental variables (e.g., climate, topography, and vegetation), we constructed a high-precision species distribution model to identify key factors influencing the habitat suitability of *L. chinensis* [13]. Model performance was evaluated using receiver operating characteristic (ROC) curves and area under the curve (AUC) values. The results of this study will provide critical insights into the potential distribution of *L. chinensis* under current environmental conditions and offer a scientific basis for its conservation and habitat management. Furthermore, the methodology and findings of this research can serve as a reference for predicting suitable habitats for other endangered species in similar ecological contexts [14].

## 2. Materials and Methods

### 2.1. Species Record

**Figure 1:**
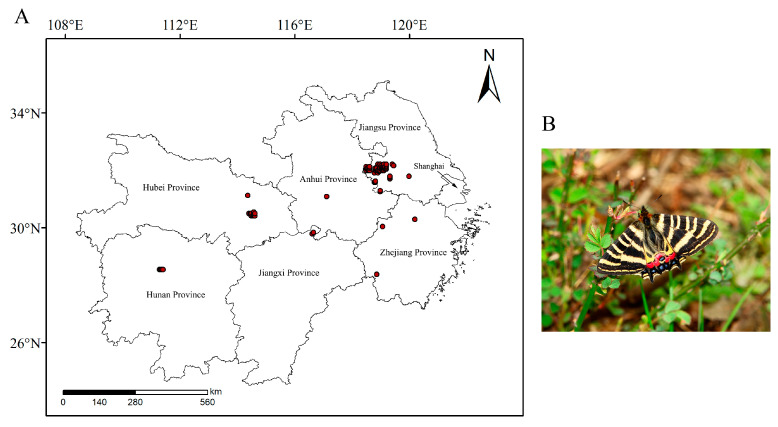
(**A**) Distribution record of the *L. chinensis* in the middle and lower reaches of the Yangtze River, (**B**) *L. chinensis*.

The study area is located in the middle and lower reaches of the Yangtze River, covering Hubei, Hunan, Jiangxi, Zhejiang, Anhui, Shanghai, and Jiangsu, with a total area of approximately 800,000 square kilometers, accounting for 8.3% of China’s total land area [15]. Species distribution records were obtained from the iNaturalist website (https://www.inaturalist.org), literature data (retrieved through Web of Science and SpringerLink databases), and the GBIF website (https://www.gbif.org), resulting in a total of 335 species distribution points with precise geographic coordinates within China. To prevent model overfitting, if the distance between two distribution points was less than the resolution of the environmental factor grid (1 km), one of the points was removed, resulting in 127 valid distribution points [16]. These points were compiled into an Excel sheet with species names, longitude, and latitude (in decimal degrees) and saved as a CSV file for MaxEnt model analysis (Figure 1).

### 2.2. Sources and Filtering of Environment Variables

This study selected 22 environmental variables, including 19 bioclimatic factors, terrain factors (aspect, elevation, slope), vegetation coverage (gm_ve), land cover (gm_lc), and human footprint (hf). The data were obtained from the WorldClim website (https://worldclim.org) [17]. Contemporary climate data (1970–2000) were downloaded from WorldClim version 2.1 at a resolution of 5 arc-minutes. Future climate scenario data were derived from the Beijing Climate Center Climate System Model (BCC-CSM2-MR) under the Sixth Coupled Model Intercomparison Project (CMIP6), also at a 5 min resolution. The future data included projections for the 2050s (2041–2060), 2070s (2061–2080), and 2090s (2081–2100). CMIP6 comprises seven Shared Socioeconomic Pathways (SSPs): SSP1-1.9, SSP1-2.6, SSP2-4.5, SSP3-7.0, SSP4-3.4, SSP4-6.0, and SSP5-8.5 [18]. This study utilized the SSP2-4.5 scenario, representing a moderate greenhouse gas emission pathway, to evaluate the potential impacts of future climate change on species distribution. To avoid multicollinearity among environmental variables, which could lead to model overfitting, Spearman’s rank correlation analysis was applied to the environmental data for de-correlation [19]. First, variables with an absolute correlation coefficient of less than 0.7 were retained from the initial 22 variables. For pairs of variables with a correlation coefficient greater than 0.7, the variable with the lower ecological relevance was removed [20]. Subsequently, a spatial cross-validation approach was employed, using the True Skill Statistic (TSS) as the evaluation metric, to further refine the variable selection after addressing collinearity. Based on the contribution of each environmental factor, 14 variables with significant statistical and biological relevance were retained as input data for model construction. These variables included bio01, bio03, bio04, bio05, bio06, bio09, bio11, bio17, elev, aspect, slope, gm_ve, gm_lc, and hf.

### 2.3. MaxEnt Model Construction and Validation

To optimize the MaxEnt model parameters, we employed the kuenm_ceval function [21], evaluating models based on statistical significance (partial receiver operating characteristic (ROC), 500 iterations), predictive ability (omission rate (OR)), and model complexity (Akaike Information Criterion corrected for small sample sizes, AICc). Following the “OR_AICc” criterion, significant candidate models were identified as those with an omission rate below the predefined threshold (e.g., ≤0.05, where applicable) and the lowest AICc value (ΔAICc ≤ 2). These models were selected as the final models with optimal parameters [22]. The MaxEnt model parameters included feature combinations (FCs), regularization multipliers (RMs), and maximum background points (BCs), among others [19]. To determine the optimal parameter combination, we systematically explored various configurations of two key parameters: feature classes and regularization multipliers [23]. Specifically, five MaxEnt feature classes generated 31 unique feature combinations, while regularization multipliers were tested at 40 values ranging from 0.1 to 4, with intervals of 0.1. This resulted in a total of 1240 candidate models being evaluated for their performance.

The receiver operating characteristic (ROC) curve was used to evaluate the accuracy of model prediction, where the area under the ROC curve (AUC) was the performance indicator of MaxEnt prediction [24]. The closer the AUC value is to 1.0, the higher the accuracy of the model, thus determining the best prediction model.

### 2.4. Habitat Classification and Centroid Method

In this study, ArcGIS 10.2 was used to classify the results of the MaxEnt model into distinct suitability levels. The natural breaks classification method was applied to categorize suitable habitats into four classes: unsuitable habitats, low-suitability habitats, medium-suitability habitats, and high-suitability habitats [25,26]. The centroid shift reflects the spatial variation of the species over a specific time period. Using the SDMtoolbox in ArcGIS, distribution maps were generated to visualize the spatial pattern changes in suitable habitats for *L. chinensis*. The geometric centroid of suitable habitats for *L. chinensis* represents its distribution center, with the centroid’s location indicating the overall spatial distribution of high-suitability areas [27]. The partition geometry statistics tool in ArcGIS was used to calculate the centroids of suitable habitats under different SSP2-4.5 climate scenarios across various time periods. Vector files depicting the changes in centroids between adjacent periods were generated to characterize the migration trend and distance of the centroid.

## 3. Results and Analysis

### 3.1. Model Prediction Evaluation and Selection of Environmental Factors

By running the kuenm package, we identified a model that satisfied both the OR and AICc criteria, and we selected M_4_F_qt_Set1 (regularization multiplier = 4, feature combination = Q and T). This study retained 14 key ecological factors influencing the distribution of *L. chinensis*, including bio17 (precipitation in the driest quarter), bio03 (isothermality), bio06 (minimum temperature of the coldest month), bio04 (temperature seasonality coefficient), bio11 (average temperature of the coldest quarter), elevation (elev), bio09 (average temperature of the driest quarter), bio05 (maximum temperature of the warmest month), bio01 (annual average temperature), aspect, slope, gm_ve (vegetation coverage percentage), gm_lc (land cover type), and hf (human activity impact index), for modeling. After inputting these factors into the MaxEnt model, the results showed an AUC value of 0.989, indicating excellent model prediction performance (Figure S1). The model, constructed based on the initial environmental factors, is suitable for simulating suitable habitats for *L. chinensis*. Subsequently, the relative importance of key climatic factors was evaluated using methods such as total contribution rate, permutation importance, and the Jackknife test.

**Figure 2:**
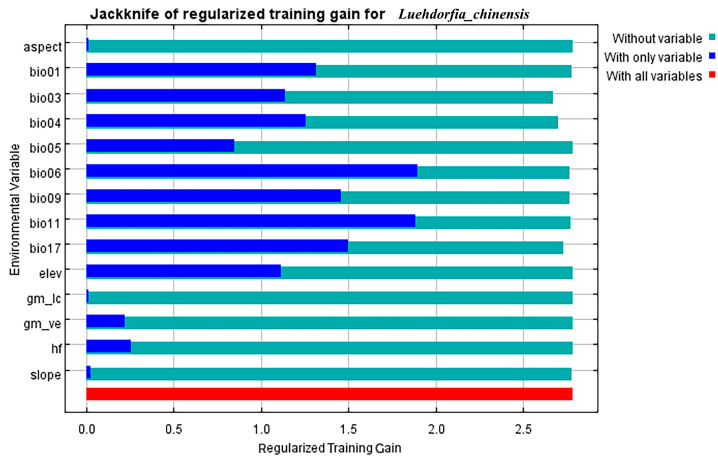
Jackknife plot of environmental factors affecting the ecological distribution of *L. chinensis*.

Using the Jackknife test in the MaxEnt model to validate the gain of environmental variables, combined with the importance test results, all environmental factors showed a high cumulative contribution rate to the model (Table 1, Figure 2). From the Jackknife plot, the three most important environmental factors for prediction were identified as bio17 (precipitation of the driest quarter), bio03 (isothermality), and bio06 (minimum temperature of the coldest month). After comprehensive analysis, the following six key environmental factors were selected: bio17 (precipitation in the driest quarter), bio03 (isothermality), bio06 (minimum temperature of the coldest month), bio04 (temperature seasonality coefficient), bio11 (average temperature of the coldest quarter), and elevation (elev). These factors meet the selection criteria of a cumulative contribution rate greater than 95% and an individual factor contribution rate of no less than 2%. The validation results show that the modeling contribution rates for the six environmental factors are 36.7%, 17.3%, 12.9%, 11%, 9.1%, and 8.4%, respectively, with a cumulative contribution rate of 95.4%. Furthermore, the correlation coefficients between the factors are all less than 0.7. Therefore, these six ecological factors are the key predictors of suitable habitats for *L. chinensis*.

The response curves reveal the relationship between the main environmental variables and suitable habitats for *L. chinensis*, helping us understand the species’ adaptability to environmental conditions (Figure S2). Based on the response curves for the key environmental factors and the logistic output, the most suitable habitat conditions for *L. chinensis* are as follows: precipitation in the driest quarter (bio17) ranges from 99.0 mm to 130.6 mm; isothermality (bio03) ranges from 24.4 to 26.2; minimum temperature of the coldest month (bio06) ranges from −1.8 °C to 0.6 °C; temperature seasonality coefficient (bio04) ranges from 902.8 to 932.8; average temperature of the coldest quarter (bio11) ranges from 3.1 °C to 5.4 °C; and elevation (elev) is 87.2 m or lower, where *L. chinensis* is more likely to thrive. Particularly, when the elevation is 69.3 m, the likelihood of survival for the butterfly is highest. Within a certain elevation range, the lower the elevation, the more suitable it is for the butterfly’s survival.

### 3.2. Distribution and Patterns of Suitable Habitats for L. chinensis

#### 3.2.1. Current Distribution of Suitable Habitats

**Figure 3:**
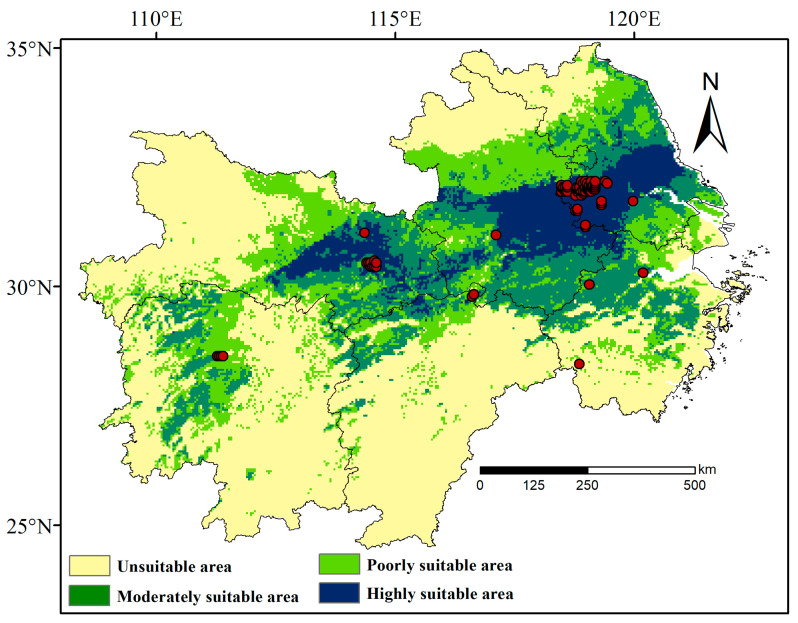
Current distribution of suitable habitats for *L. chinensis* in the lower and middle Yangtze River Basin. The red circles represent the distribution points of *L. chinensis*.

Under current climate conditions (Figure 3), the highly suitable habitats for *L. chinensis* are primarily distributed around Wuhan and Xiaogan in Hubei Province, with additional distribution in Anhui and Jiangsu Provinces. The total area of this region is 75.1 × 10^3^ km^2^, accounting for 8.8% of the total area of the lower and middle Yangtze River Basin. The area of moderately suitable habitats is 146.6 × 10^3^ km^2^, accounting for 17.2% of the region, primarily distributed in parts of Hunan, Jiangxi, Hubei, and Zhejiang Provinces. The area of low-suitability habitats is 173.1 × 10^3^ km^2^, covering 20.4% of the total area, and is mainly concentrated in Hubei, Hunan, Anhui, and Jiangsu Provinces. The highest suitability is concentrated in core zones (dark shading), with a progressive decline toward peripheral areas (lighter shading).

#### 3.2.2. Future Predictions of Suitable Habitats

Under the SSP2-4.5 climate scenario, the change in highly suitable habitats is significant (Table 2). By 2041–2060, the area of highly suitable habitats will reach 26.4 × 10^3^ km^2^, concentrated in central Anhui and central Jiangsu, accounting for 3.1% of the total area of the lower and middle Yangtze River Basin, a reduction of 64.8% compared to the current area. By 2061–2080, the area of highly suitable habitats will be approximately 52.5 × 10^3^ km^2^, covering 6.2% of the total area, a decrease of 30.1% compared to the present. During this period, the highly suitable habitats will be primarily distributed in Anqing, Anhui, Xiaogan, Hubei, and Nanjing, Jiangsu. By the 2090s, the highly suitable habitats will significantly shrink to 8 × 10^3^ km^2^, covering only 0.9% of the total area of the lower and middle Yangtze River Basin, a decrease of 89.3% compared to the current area. These habitats will be primarily found in Jiangxi and Jiangsu provinces, showing a clear declining trend. In contrast, the area of low-suitability habitats will change more steadily. By the 2070s, the area of low-suitability habitats will reach 283.4 × 10^3^ km^2^, accounting for 33.3% of the total area of the lower and middle Yangtze River Basin, an increase of 63.7% compared to the current area. By the 2090s, the change in the area of low-suitability habitats will be relatively small, covering 20.5% of the total area, with a slight increase of 0.6%. The area of moderately suitable habitats shows varying trends over different time periods. In the 2070s, it is expected to decrease by 22.2%, covering 13.4% of the study area. By the 2050s, the change in moderately suitable habitats will be relatively small, decreasing by approximately 2%, reaching 143.7 × 10^3^ km^2^, which accounts for 17.2% of the total area. Under the future SSP2-4.5 scenario, it is expected that the area of unsuitable habitats will significantly expand, while the area of highly suitable habitats will drastically shrink. Much of the Hunan and Jiangxi provinces will no longer be suitable for the survival of *L. chinensis*, and significant areas of unsuitable habitats will also emerge in provinces such as Hubei and Zhejiang.

**Figure 4:**
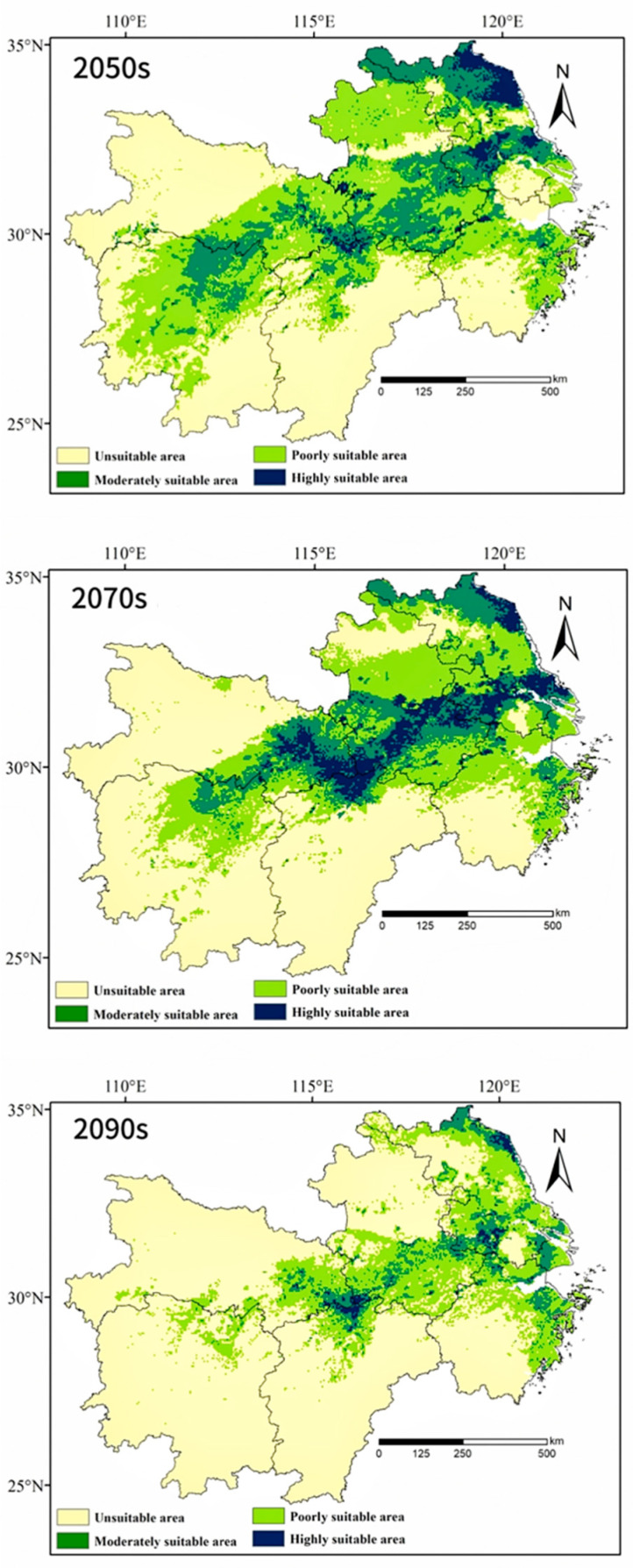
Distribution of *L. chinensis* in the lower and middle Yangtze River Basin.

Overall, in the future, most areas, except for those along the Yangtze River and its tributaries, will become unsuitable for the survival of *L. chinensis*. This indicates that the population of the species will face significant challenges to persistence. Therefore, it is crucial to strengthen the protection and management of its habitats (Figure 4).

### 3.3. Centroid Migration of Suitable Habitats for L. chinensis

**Figure 5:**
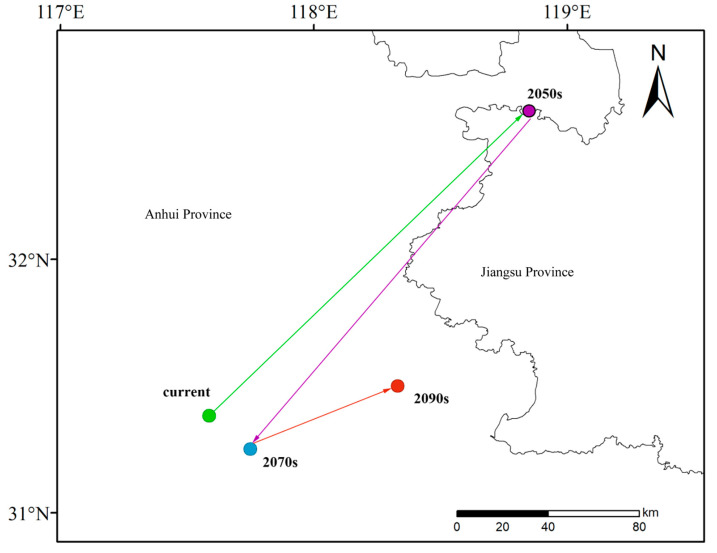
Centroid displacement of *L. chinensis* at different periods.

Under the current climate conditions, the centroid coordinates of *L. chinensis* are 117°35′7.6128″ E, 31°23′0.1176″ N, located in Dingyuan County, Chuzhou City, Anhui Province. Under the moderate greenhouse gas emission scenario SSP2-4.5, in the 2050s, the centroid of *L. chinensis* will gradually shift northeast by 47.1° towards the direction of 47.1°, moving 176.23 km to Gaochun District, Nanjing City, Jiangsu Province, with specific coordinates at 118°50′55.9284″ E, 32°35′5.5716″ N. In the 2070s, the centroid will shift southwest by 34.9°, moving 164.02 km back to Dingyuan County, Chuzhou City, Anhui Province, with specific coordinates at 117°44′55.4028″ E, 31°15′1.9548″ N. By the 2090s, the centroid will again shift northeast by 80.16°, moving 61.48 km, returning once again to Chuzhou City, Anhui Province, with coordinates at 118°19′48.828″ E, 31°29′57.5772″ N (Figure 5, Table 3).

## 4. Discussion

The *L. chinensis* is a rare and endemic insect species in China, relying on various host plants and nectar plants for its growth. To accurately predict its suitable habitat range, this study screened distribution point data in ENMTools software (ver 1.31) and applied the “Jackknife test” combined with correlation analysis to remove environmental variables with a correlation coefficient greater than 0.7 [20,28], in order to avoid model overfitting due to collinearity. The optimized model achieved an AUC value of 0.989, with the ROC curve approaching the top-left corner, indicating that the model has high predictive accuracy and reliability [27,28,29,30]. By constructing the MaxEnt model, this study was able to assess the habitat suitability of *L. chinensis* in different regions, predict its potential distribution, and identify the key environmental factors influencing its distribution. Based on the model results, the existing protected area layout can be optimized, and the protection range can be expanded, helping to identify the most suitable areas for the species’ survival and reproduction. This also provides insights into how the species responds to climate change and habitat alterations. The findings provide scientific support for establishing and optimizing protected areas, which is of significant conservation value [31]. The MaxEnt model not only provides decision support for the construction and management of protected areas but also helps address challenges such as climate change, thereby promoting the conservation of biodiversity.

This study, based on model analysis, demonstrates the potential suitable habitat distribution of *L. chinensis* in the Yangtze River Basin and explores the environmental factors influencing its distribution. This study shows that the primary environmental factors affecting the distribution of *L. chinensis* include Bio17 (precipitation in the driest quarter), bio03 (isothermality), bio06 (minimum temperature of the coldest month), bio04 (temperature seasonality), bio11 (mean temperature of the coldest quarter), and elev (elevation). Among these, temperature and precipitation have the greatest impact on its distribution. The driest quarter precipitation (bio17) contributes the most to the model, accounting for 36.7%. When the precipitation in the driest quarter ranges between 99.0 mm and 130.6 mm, it is most favorable for the survival of *L. chinensis*. *Asarum* species are its main host plants [32], which thrive in shady, moist environments; excessive or insufficient precipitation can negatively impact plant growth, thereby affecting the survival of the butterfly [33]. Temperature is also an important factor influencing the distribution of *L. chinensis*, with the environmental factors related to temperature contributing 50.3% in this study. Temperature not only affects the growth and distribution of the butterfly but also significantly impacts its habitat. Specifically, it influences processes such as seed germination, fruit maturation, and plant metabolism. As temperature plays a critical role in shaping the overall ecosystem, it directly affects the availability of suitable host plants, which in turn impacts the butterfly’s survival and reproduction. Temperature changes may also disrupt the synchronized growth relationship between *L. chinensis* and its host plants, affecting its reproduction and larval survival. As global temperatures rise, climate change in the middle and lower Yangtze River region is having a significant impact on suitable habitats for *L. chinensis*, leading to its migration. This disruption in ecological relationships could cause shifts in population dynamics, making it essential to monitor and manage both climate and habitat changes to ensure the species’ survival. The migration trend is evident in the model’s predictions, suggesting that, with ongoing climate change, the butterfly’s potential distribution areas may shift, thereby presenting challenges for its conservation [34]. The study found that *L. chinensis* thrives when the average temperature in the coldest quarter (bio11) is between 3.1 °C and 5.4 °C and the minimum temperature in the coldest month (bio06) falls between −1.8 °C and 0.6 °C. Although the effect of altitude on its distribution is relatively small (contribution rate of 8.4%), it remains significant. Lower altitudes support the survival of *L. chinensis*, as higher altitudes are typically characterized by stronger sunlight, lower oxygen levels, and higher atmospheric pressure, all of which reduce the butterfly’s chances of survival [35]. Vegetation coverage has a relatively minor impact on the distribution of *L. chinensis*, with environmental factors such as temperature and precipitation playing a more critical role. Furthermore, under the moderate greenhouse gas emission scenario (SSP2-4.5), this study predicts an overall decrease in the butterfly’s suitable habitat area. By the 2090s, the area of high suitability, primarily concentrated in central Anhui Province, will shrink by 89.3%, while the area of moderate suitability will decrease by 65.1%. The area of low suitability will change minimally, while the area unsuitable for habitation will increase significantly, reaching 616.8 × 10^3^ km^2^, or 72.6% of the total area of the lower Yangtze River region [36]. Additionally, the geometric centroid of *L. chinensis* will gradually shift toward the northeast [36].

This study mainly focuses on factors such as temperature, precipitation, altitude, and slope. While human activities may have limited direct impact on climatic factors such as temperature and precipitation, their destructive effects on the habitat of *L. chinensis*, including deforestation and land cover changes, cannot be overlooked. The accelerated urbanization process in the lower Yangtze River region has led to significant deforestation, while increased soil erosion has further altered land cover types, thereby disrupting plant growth and distribution. This, in turn, affects the availability of food sources and suitable habitats for *L. chinensis*. These anthropogenic factors may exacerbate the threats faced by the butterfly species [37]. This research only uses the MaxEnt model to analyze the potential distribution of *L. chinensis* under the SSP2-4.5 climate scenario, without addressing the future distribution of its host plants or natural predators. Future studies should expand the scope to accurately simulate more comprehensive distribution areas, incorporating additional environmental and biological factors to improve the accuracy and reliability of the results. This would provide more targeted scientific support for the conservation of *L. chinensis* and its habitat.

## 5. Conclusions

This study utilized the MaxEnt model to predict the future suitable distribution areas of *L. chinensis* (Chinese butterfly) under the medium greenhouse gas emission scenario SSP2-4.5. The model’s accuracy was confirmed with an AUC value of 0.989, indicating highly reliable predictions. Subsequently, the contribution of each environmental factor was analyzed using the Jackknife test, revealing that precipitation and temperature are the two most influential factors on the butterfly’s distribution, with temperature having the greatest contribution. Under the SSP2-4.5 climate scenario, it was predicted that, by the 2090s, the geometric centroid of *L. chinensis* will shift northeastward. Moreover, compared to the present period, the area of non-suitable habitats will dramatically increase to 616.8 × 10^3^ km^2^, accounting for 72.6% of the total area in the lower Yangtze River region. These results indicate that, with the significant reduction in high-suitability habitats and the sharp expansion of non-suitable areas, *L. chinensis* will face severe challenges for survival in China in the future. To address this challenge, urgent and scientifically informed conservation measures are required. These include conducting research on genetic diversity in suitable habitats, enhancing conservation efforts, and creating appropriate environments to ensure the survival and reproduction of *L. chinensis*.

## Figures and Tables

**Table 1:** Contribution values and permutation importance of key environmental factors.

Environment Variable	Description	Contribution Rate	Permutation Importance
bio17	Precipitation in the driest quarter	36.70%	3.70%
bio03	Isothermal property	17.30%	46.80%
bio06	Minimum temperature in the coldest month	12.90%	17.80%
bio04	Coefficient of seasonal variation of temperature	11%	7.60%
bio11	Mean temperature of coldest quarter	9.10%	1.50%
elev	Altitude	8.40%	0.20%
bio09	Mean temperature of driest quarter	1.90%	16.60%
bio05	Max temperature of warmest month	1.70%	1.60%
bio01	Annual mean temperature	0.50%	3.10%
aspect	Aspect	0.20%	0.00%
slope	Slope	0.20%	0.70%
gm_ve	Vegetation coverage percentage	0.10%	0.20%

**Table 2:** Comparison of suitable habitat areas across different periods under the SSP2-4.5 climate scenario with the current period (10^3^ km^2^, %).

Predicted Area (10^3^ km^2^)					Comparison with Current Distribution (%)			
Period	Unsuitable	Poorly Suitable	Moderately Suitable	Highly Suitable	Unsuitable	Poorly Suitable	Moderately Suitable	Highly Suitable
Current	455.2	173.1	146.6	75.1	——	——	——	——
2050s	396.6	283.4	143.7	26.4	−12.9	63.7	−2	−64.8
2070s	465.2	218.3	114	52.5	2.2	26.1	−22.2	−30.1
2090s	616.8	174.1	51.1	8	35.5	0.6	−65.1	−89.3

**Table 3:** Centroid displacement angle, direction, and distance of *L. chinensis* under the SSP2-4.5 climate scenario across different periods (° and km).

Scene	Period	Angle/°	Direction	Displacement/km
	Present to 2050s	47.1	Northeast	176.23
SSP2-4.5	2050s to 2070s	235.1	Southwest	164.02
	2070s to 2090s	80.16	Northeast	61.48

## Data Availability

The data supporting the results are available in a public repository at GBIF.org; GBIF Occurrence Download is available at https://doi.org/10.15468/dl.4gyzgp; and *L. chinensis* occurrence data are available at 10.6084/m9.figshare.28089503.

## Supplementary Materials

The following supporting information can be downloaded at https://www.mdpi.com/article/10.3390/insects16040396/s1, Figure S1: The receiver operating characteristic (ROC) curve and the area under the curve (AUC) 162 simulated in the potential distribution area of *L. chinensis*; Figure S2: Response curves of environmental variables in the MaxEnt model, illustrating 195 their influence on habitat suitability for *L. chinensis*.
