# Supplementary material for: Habitat Suitability Analysis for Luehdorfia chinensis Leech, 1893 (Lepidoptera: Papilionidae) in the Middle and Lower Yangtze River: A Study Based on the MaxEnt Model
Source: Insects. 2025 Apr 9;16(4):396. doi: 10.3390/insects16040396 (PMC12027586; doi:10.3390/insects16040396)
Supplement: Supplementary file 1 [file insects-16-00396-s001.zip › insects-3525494-supplementary.pdf]

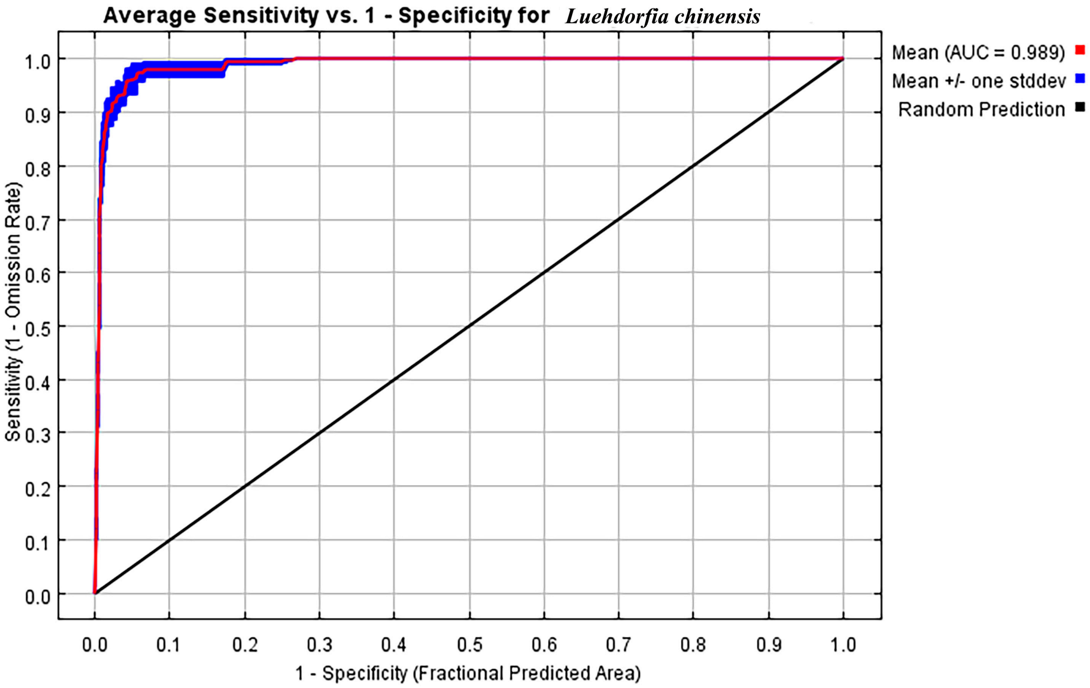

Figure S1. The receiver operating characteristic (ROC) curve and the area under the curve (AUC) 162 simulated in the potential distribution area of *L.chinensis*.

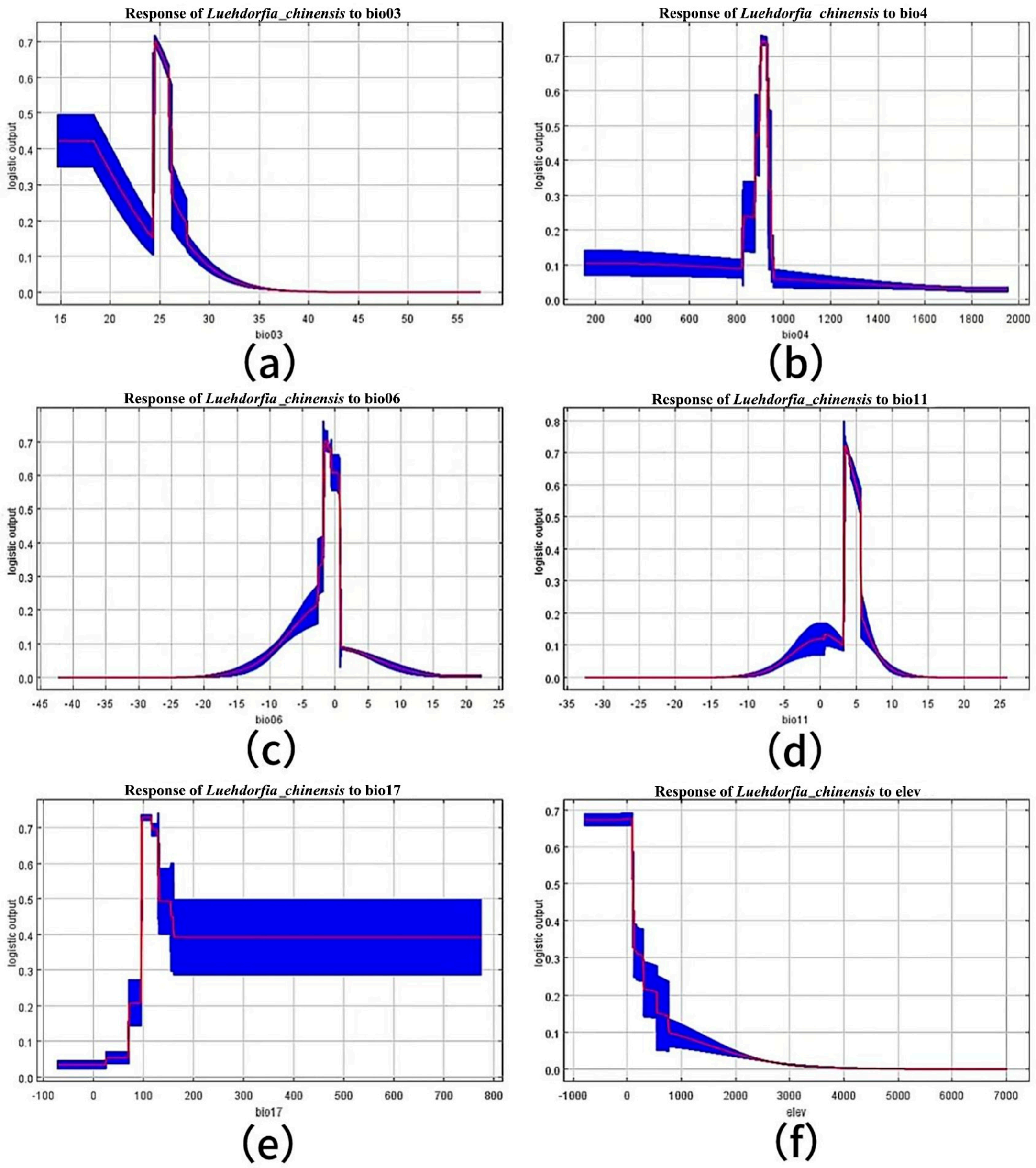

Figure S2. Response curves of environmental variables in the MaxEnt model, illustrating their influence on habitat suitability for *L.chinensis*.
