# Supplementary figures and images for: Habitat Suitability Analysis for Luehdorfia chinensis Leech, 1893 (Lepidoptera: Papilionidae) in the Middle and Lower Yangtze River: A Study Based on the MaxEnt Model
Source: Insects. 2025 Apr 9;16(4):396. doi: 10.3390/insects16040396 (PMC12027586; doi:10.3390/insects16040396)

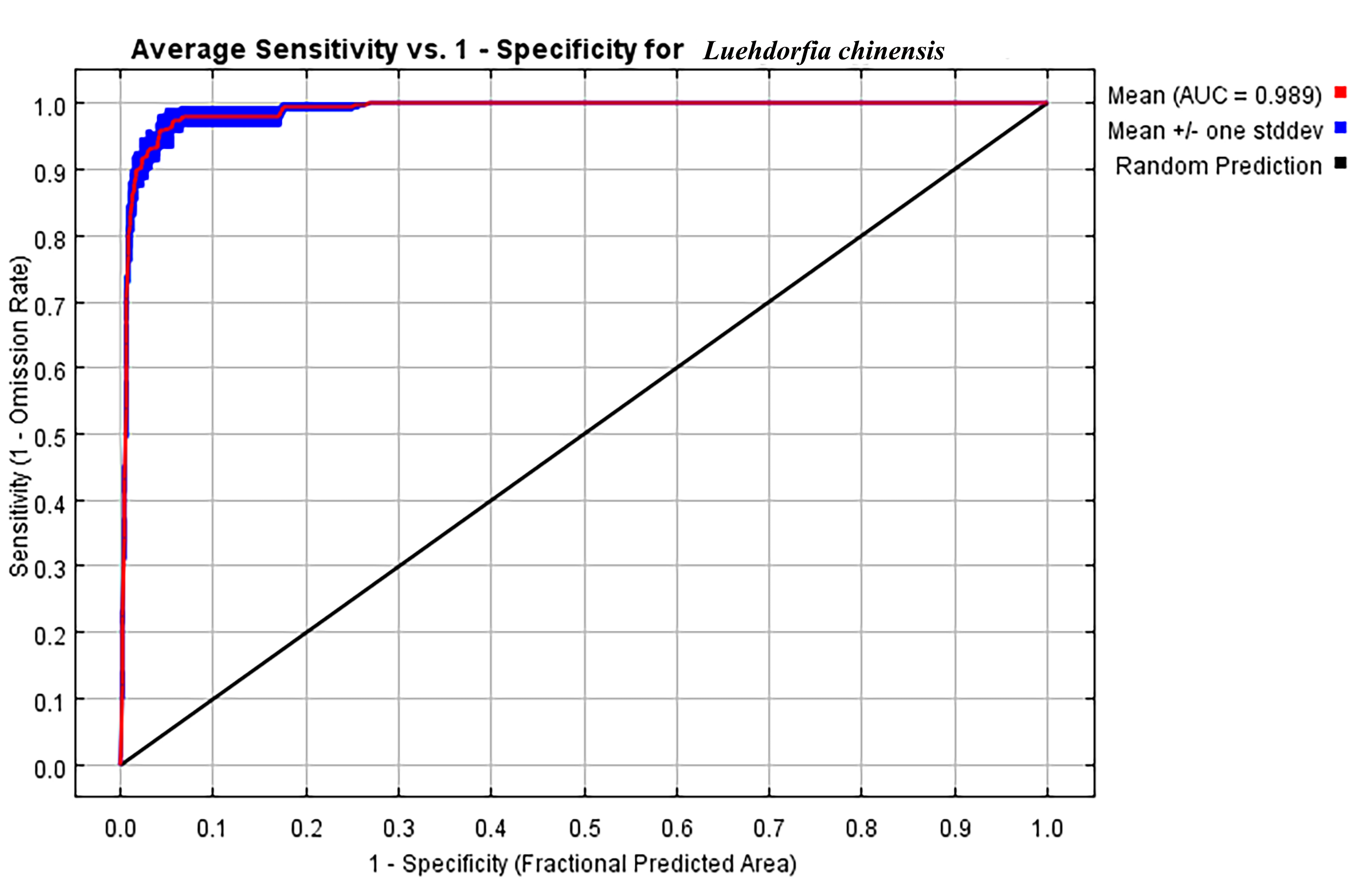

Supplement: Supplementary file 1 [file insects-16-00396-s001.zip › Figure S1.tif]

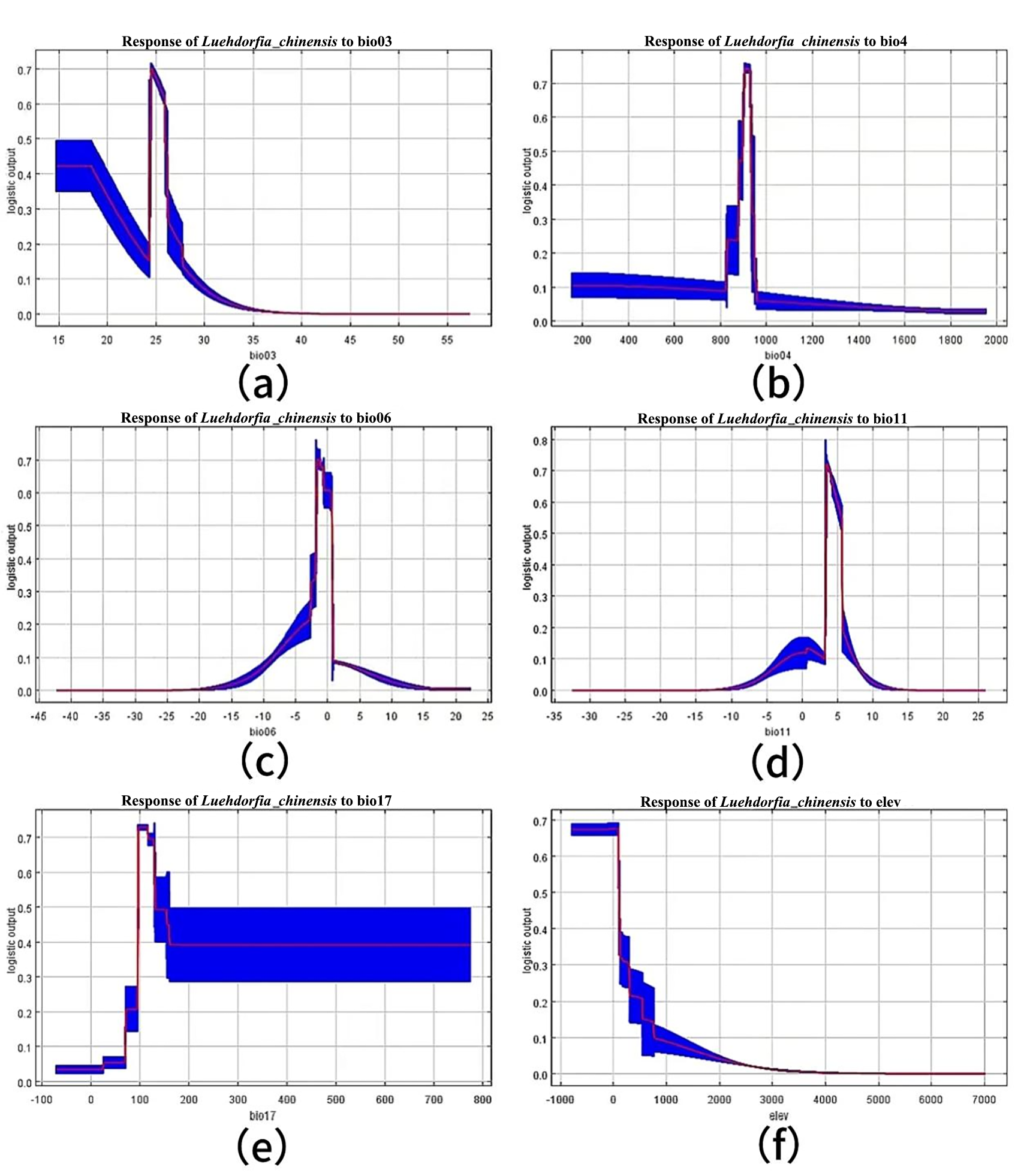

Supplement: Supplementary file 1 [file insects-16-00396-s001.zip › Figure S2.tif]
